# Indigenous university students' perceptions regarding nature, their daily lives and climate change: a photovoice study

**DOI:** 10.1186/s12889-024-21111-6

**Published:** 2025-01-08

**Authors:** Ieda M. A. V. Dias, Antonio Jose Grande, Paulo T. C. Jardim, Alessandra Aparecida Vieira Machado, Jacks Soratto, Maria Inês da Rosa, Luciane Bisognin Ceretta, Leonardo Roever, Xanthi Zourntos, Seeromanie Harding

**Affiliations:** 1 Federal University of Rio Grande Do Sul, Porto Alegre, Rio Grande Do Sul Brazil; 2 Medicine School, State University of Mato Grosso Do Sul, Campo Grande, Mato Grosso Do Sul Brazil; 3 Public Health Department, Universidade Do Extremo Sul Catarinense, Santa Catarina, Brazil; 4 Department of Clinical Research, Brazilian Evidence-Based Health Network, Uberlândia, 38408-100 Brazil; 5 Gilbert and Rose-Marie Chagoury School of Medicine, Lebanese American University, Beirut, 1401 Lebanon; 6 Department of Population Health Sciences, School of Life Course & Population Sciences, King’s College London, Franklin-Wilkins Building, Stamford Street London, SE1 9NH UK

**Keywords:** Indigenous, Climate change, Community-based participatory research, Documentary photography, Local knowledge

## Abstract

**Background:**

Climate change has severe health impacts, particularly for populations living in environmentally sensitive areas such as riversides, slopes, and forests. These challenges are exacerbated for Indigenous communities, who often face marginalisation and rely heavily on the land for their livelihoods. Despite their vulnerability, the perspectives of Indigenous populations on climate change and its impacts remain underexplored, creating a critical gap in the literature. This study explored the perceptions of Indigenous Brazilian university students on how climate change affects their daily lives and gathered their insights on potential adaptations to mitigate climate change-related impacts.

**Methods:**

Using a participatory arts-based approach, participants captured photographs reflecting their lived experiences with climate change. Follow-up interviews provided a narrative framework for qualitative analysis, enabling participants to articulate the strengths and concerns of their communities while transcending cultural and linguistic barriers.

**Results:**

The study revealed key themes, including (1) the fragility of ecosystems critical to Indigenous livelihoods, (2) the erosion of traditional knowledge systems due to environmental and social disruptions, and (3) the need for community-driven strategies to protect territories and preserve cultural identities. Participants highlighted the interconnectedness of their cultural values with environmental stewardship, emphasising the importance of maintaining these relationships as a form of resilience.

**Conclusion:**

This study underscores the importance of protecting Indigenous territories and respecting their cultural identities to safeguard their survival and traditions. The voices of Indigenous university students provided valuable insights into community-based adaptations and strategies for mitigating the impacts of climate change.

## Introduction

Climate change is a global issue impacting all of humanity, but its effects are particularly detrimental to Indigenous peoples in Brazil, who have historically relied on natural resources for their survival and cultural practices. Despite efforts by governmental and non-governmental organizations to address climate change, challenges persist in tackling environmental degradation, rising global temperatures, the increasing frequency of extreme weather events, and their inequitable impacts [1]. The World Health Organization (WHO) has identified climate change as the most significant threat to global health systems in the twenty-first century [2].

The health impacts of climate change are disproportionately severe for populations in environmentally sensitive areas such as riverbanks, slopes, and forests. This vulnerability is further compounded for those whose livelihoods depend on the land and for communities experiencing inequality, marginalization, and colonization—such as Indigenous peoples [3]. These communities rely on local biodiversity and ecosystems for their well-being and survival. However, they face five significant risks in the context of climate change, as outlined by the International Labour Organization (ILO): poverty, dependence on renewable natural resources vulnerable to climate change, high migration rates due to environmental changes, gender inequality, and exclusion from decision-making processes about their rights. These intersecting risks limit their capacity to adapt to or mitigate the effects of climate change [4].

Indigenous peoples possess extensive environmental knowledge passed down through generations, which is a critical resource for climate change adaptation. This traditional knowledge enables Indigenous communities to survive adverse conditions, manage natural resources sustainably and maintain a worldview deeply intertwined with environmental stewardship. The Latin America and the Caribbean (LAC) region faces fragile economies and healthcare systems, and Indigenous peoples— over 40 million in size—are particularly at risk. The intricate connection between their health and ecosystems makes climate change a profound public health threat in these regions [5].

In Brazil, the impact of climate change is starkly evident. Satellite image data from 1985 to 2020 reveal that an average of 150,957 km^2^ of land—approximately 1.8% of the country's territory—was affected by fires annually, with 11.2% of these burned areas located in Indigenous territories [6]. These fires represent only one facet of climate change related impacts; water resources are also increasingly strained by scarcity and contamination. Deforestation exacerbates these challenges, reducing vegetation cover and contributing significantly to carbon emissions. Brazil, home to the world's second-largest vegetation cover after Russia, is a major contributor to global emissions, ranking fourth when deforestation-related CO2 emissions are included [7]. Despite these challenges, Indigenous peoples in Brazil remain at the forefront of land and cultural preservation. Since the end of Brazil’s dictatorship in 1985, they have gained significant political rights, culminating in the establishment of the Ministry of Indigenous Peoples in 2023. Indigenous groups in the Amazon play a crucial role in global climate mitigation, having conserved a carbon stock equivalent to 114 million trees, according to the Raízes do Purus project [8].

This study uses photovoice to explore the perceptions of Indigenous Brazilian university students regarding the impacts of climate change on their daily lives. To address the upskilling of Indigenous communities, Brazil provides opportunities for culturally adapted university level training for Indigenous young people from the Indigenous Lands. These young people have lived experience of impact of climate related impacts on their communities. They are also a powerful young voice of their communities. The study gathers their insights on potential community-driven strategies to mitigate or prevent these impacts, highlighting the intersection of traditional knowledge and lived experiences.

## Methods

### Methodology and research framework

Photovoice is an arts-based participatory method that engages participants throughout every stage of the research process, fostering mutual trust and respect while building the capacity of participants' photovoice research skills. This methodology empowers participants to visually express their perspectives, ensuring their genuine involvement in the co-production of knowledge [9, 10]. By identifying, representing, and strengthening their communities through photography, photovoice promotes collaboration between participants and researchers, fostering a nuanced understanding of shared challenges [11–14].

### Study design

**Fig. 1:**
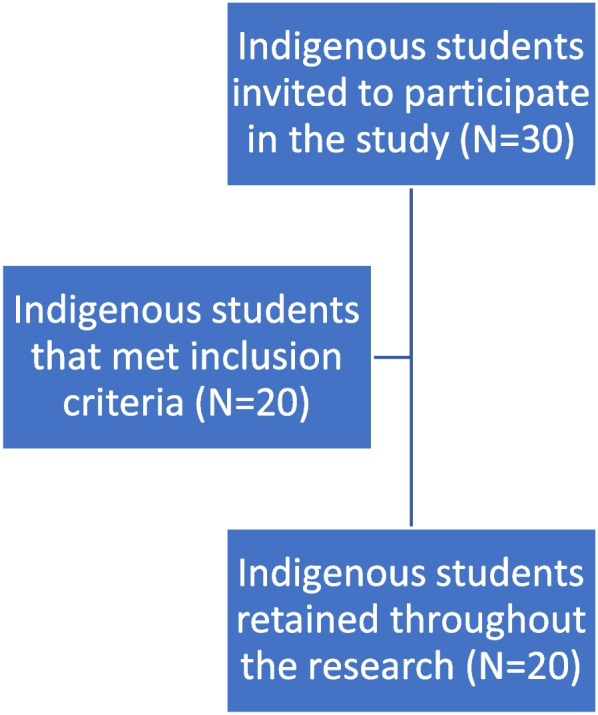
Flowchart of the study participants

Participants captured photographs that reflected their experiences, concerns and perspectives. In follow-up interviews, participants presented their images and shared their reflections, explaining the context, motivation, and significance behind each photograph. These photographs, coupled with the interview narratives, served as the primary data for qualitative analysis. By integrating visual and verbal expressions, this participatory arts-based approach transcended cultural and linguistic barriers, empowering Indigenous participants to articulate the strengths and challenges within their communities [15]. Figure 1 shows the flowchart of the study participants.

### Ethics approval

Ethical considerations related to local ethics guidelines for Indigenous populations. The study adhered to the National Research Ethics Committee guidelines and was approved by Fundação Oswaldo Cruz—Brasília (Opinion No. 4,279,173). Written informed consent was obtained from all participants, who also signed photo release forms authorizing the use of their images for research purposes. Participation was voluntary, and risks were clearly outlined during an introductory meeting.

### Participants and recruitment

Participants recruited were based at universities of the researchers. Some would been selected for a scholarship based on their capabilities to complete a degree in their chosen area and as likely young Indigenous leaders. Twenty Indigenous university students from Mato Grosso do Sul and Rio Grande do Sul were invited to participate, with recruitment facilitated by university networks and community connections. The purposive selection prioritised diversity in gender, ethnicity, and from a rural or urban village to ensure a range of perspectives.

Participants aged 18 to 30 years (mean age: 25.25 years), included 12 women and 8 men, with ethnicities spanning Kaingang, Pitaguary, Terena, Atikum, Arapium, and Tabajara. Most participants (65%) received university scholarships, while others relied on parental support or had no financial assistance. Table 1 outlines the socio-demographic composition of the 20 students involved in the study, who were enrolled at the State University of Mato Grosso do Sul and the Federal University of Rio Grande do Sul.

**Table 1:** Demographic characteristics of the stakeholders

**Age**	**Mean**	**Standard deviation**	
	25.250000	4.635461	
**Gender**		**N**	**%**
	Male	8	40
	Female	12	60
**Marital Status**	Single	18	90
	Married	2	10
**Ethnicity**	Kaingang	10	50
	Pitaguary	1	5
	Terena	3	15
	Atikum	4	20
	Arapium	1	5
	Tabajara	1	5
**Income type**	Scholarship	13	65
	Parents’ help	3	15
	Self-financed	4	20
**Indigenous**	Urban	8	40
	Rural	12	60

### Photovoice protocol

The study used five structured online workshops conducted over six months via Google Meet due to COVID-19 restrictions. Workshops aimed to build trust, enhance technical skills, and guide participants in visually representing their experiences with climate change.

### Workshop details

- **Workshop 1:** Focused on introductions, establishing trust and familiarising participants with the photovoice methodology. Participants engaged in exercises selecting images that represented their emotions and preferences, fostering a friendly atmosphere.
- **Workshop 2:** Enhanced participants' technical photography skills, including framing and focus. Participants were encouraged to capture images representing their daily lives, preparing for the following sessions.
- **Workshop 3:** Developed narrative and photograph captioning skills, exploring how captions influence image interpretation. Participants selected photographs depicting "A Day in My Life" and collaboratively created captions using various writing styles.
- **Workshop 4:** Focused on research questions. Prompts included:
  - ◦ *"How do I feel/see the impacts of climate change in my daily life and community?"*
  - ◦ *"What adaptations are necessary to address these impacts?"*

Participants returned to their communities during university vacations to capture relevant images.

- **Workshop 5:** Facilitated the sharing and discussion of participants’ photographs. Minority views were ensured by voting on the most representative images, with follow-up discussions to achieve consensus.

### Data analysis

Data analysis followed Braun and Clarke's six-phase thematic analysis approach [16]:

1. **Familiarisation with data:** Researchers immersed themselves in transcripts, photographs, and captions to identify initial patterns.
2. **Initial coding:** Using color-coded labels, recurring patterns were identified across the dataset.
3. **Theme identification:** Conceptual maps were created to organise codes into broader themes.
4. **Theme refinement:** Themes were reviewed to ensure coherence and relevance.
5. **Theme definition:** Final definitions and names for themes were developed collaboratively with participants.
6. **Report production:** Participants provided feedback on findings, ensuring their perspectives were accurately represented in the final analysis.

Interviews and participant narratives played a central role, providing context and depth to the visual data. Collaboration with participants during thematic development ensured authenticity and cultural relevance.

### Public engagement

Study results were shared with participants through an online feedback session where they discussed and voted on the photographs that best represented their perspectives. Follow-up meetings facilitated consensus on the selected images. Additionally, participants were encouraged to share these findings with their communities and through local advocacy initiatives, leveraging the visual narratives for broader engagement and awareness.

## Results

The photographs shared and discussed among the students illuminated their experiences of the spaces they inhabit, their daily surroundings, and their connections to nature and land. They also reflected the forced adaptations they have made in response to climate change and environmental degradation caused by activities such as mining and illegal logging.

The narratives emphasised critical issues, including the right to self-identify as Indigenous, respect for their ways of life, cosmovision, spirituality, customs, traditions, and forms of social, economic, and political organization. They also highlighted the transmission of knowledge, institutions, practices, beliefs, and values. From these discussions, we constructed three analytical categories: (1) Protection of Indigenous Lands: tensions between demarcated territory and Indigenous land; (2) Peoples' Actions and the Voice of Nature; and (3) Ancestry Protection, Knowledge, and Resistance of Indigenous Culture.

1. Protection of Indigenous Lands: tensions between demarcated territory and Indigenous land

**Fig. 2:**
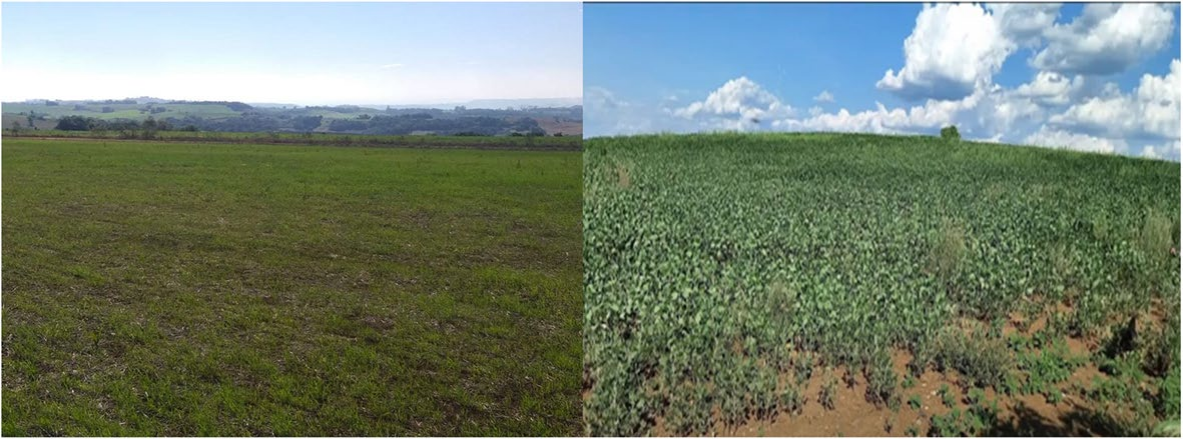
Lands leased to agribusinesses (soy production)

When discussing issues related to "Indigenous Land," participants highlighted two important aspects: the significance of Territory to Indigenous Peoples and the pressure to lease their lands. Students reported that Indigenous communities, particularly in the southern and Midwest regions of the country, are often pressured to lease the land they inhabit to agribusinesses. Figure 2 illustrates the extensive land used by agribusiness in Indigenous villages. It is important to note that leasing Indigenous Lands is prohibited in Brazil, as these lands are considered state property designated for the exclusive use of Indigenous Peoples.

The students reported that, despite a strong awareness of the prohibition against leasing Indigenous lands and the environmental harm caused by agribusiness, many Indigenous communities chose to lease their lands due to the income it provided for their survival.

"Sometimes it is necessary to lease our land, but when we accept it, we cannot forget that we will no longer be given food, we will get sick from the pesticides, everything around us will be destroyed and we will have to leave for urban centres." (Id7).

"Many of the Indigenous Lands today are leased, but it's not because we want to, we live with little, but sometimes we don't even have the little, so renting the land helps." (Id 16).

The leasing of Indigenous lands is considered a sensitive topic for several reasons, including its illegality, its destructive impact on nature, and the increase in inequalities it fosters. Additionally, it can create internal conflicts within communities when there is a lack of consensus about land leases. For instance, one participant noted that many villages have become divided due to financial proposals from those interested in leasing land. While some community members advocated for the preservation of nature, external pressures often compelled others to prioritise financial needs, despite the detrimental effects of leasing on deforestation, land depletion, and pollution.

"This question is very difficult, because there is also a fight in the village, some want it and others don't, those who want to rent are those who will have more benefits, but they don't think that today's gain can be very expensive down the road for everyone." (Id 4).

"Just to get more money, we see our houses surrounded by large crops, and when it's time to spray poison, it's horrible, we inhale everything because the poison has no borders, besides everything that is planted near these crops dies". (Id12).

**Fig. 3:**
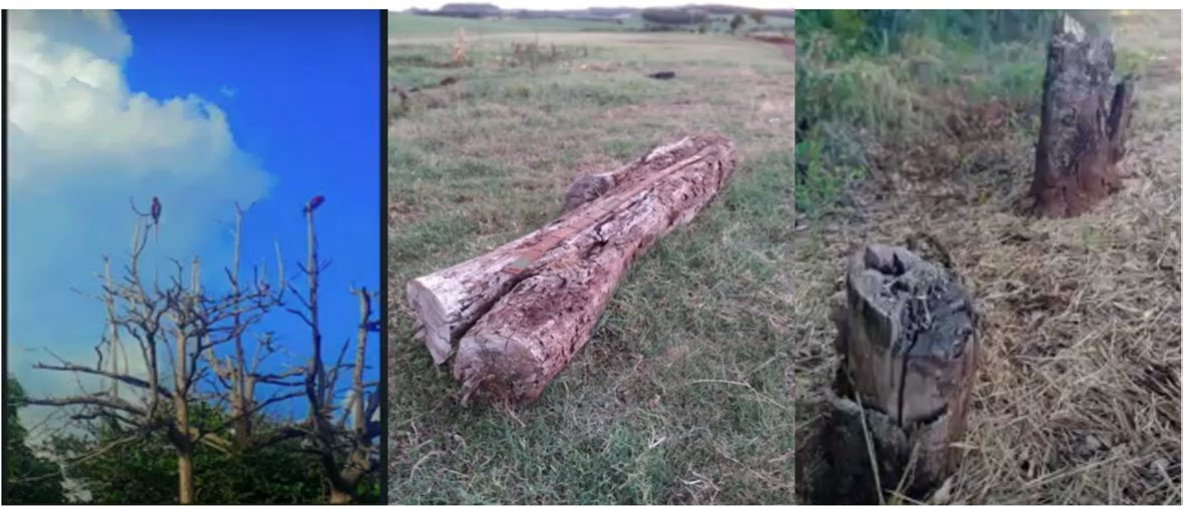
Destruction of nature

Indigenous epistemologies and worldviews regard nature as sacred territory essential for their survival. The photographs in Fig. 3 illustrate the destruction of nature resulting from the leasing of Indigenous Lands and served as a focal point for group discussion. This dialogue provided valuable insights relating to the perceptions of land held by Indigenous and non-Indigenous peoples.

"The land feeds us and gives us life, it is from which we take all the things we need. It is our great mother, and we only need a small piece of land, to fish, plant and feed ourselves, we don't need huge crops. People don't need that much to survive." (Id 9).

"We have a relationship of love and respect with the territory we occupy because, for us, the land is sacred. We come from a culture that grows for our own consumption, that without harming nature we take what we need to survive, but they don't, they just want to suck, suck and suck." (Id 12).

**Fig. 4:**
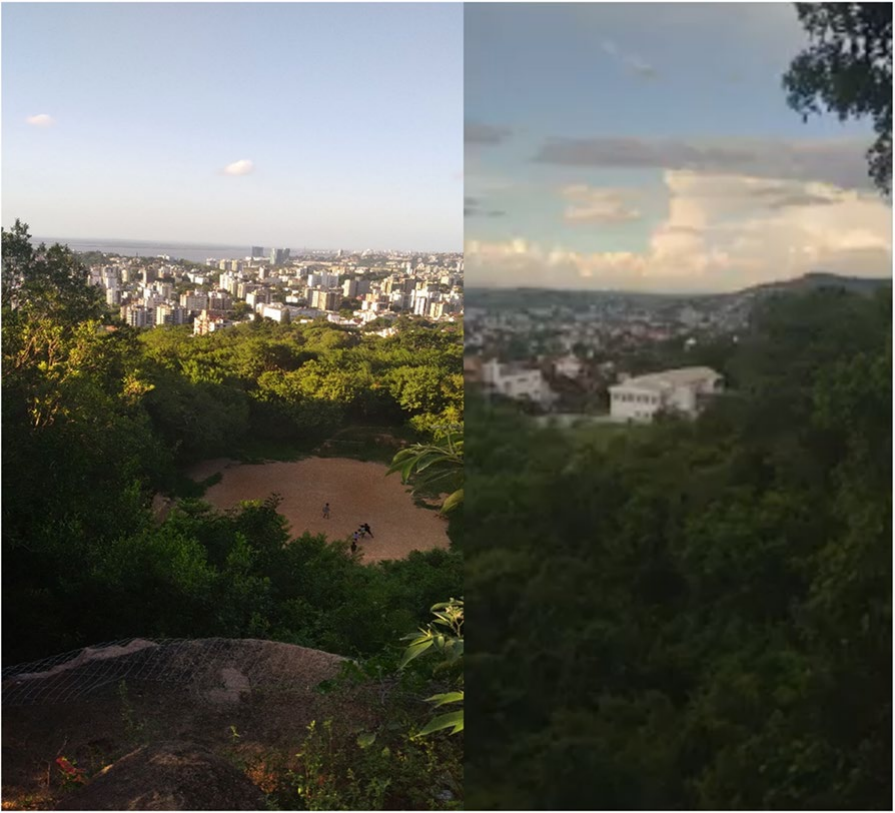
Among the city, nature fights back

While analysing these photos, participants engaged in critical reflection on the issues related to their territories and the strength derived from their connection to nature. For them, this connection fostered a profound sense of belonging. Figure 4 illustrates how aspects of their identity and cultural integrity are rooted in their relationship with nature. Participants expressed feeling empowered when viewing this image, as it symbolised the resilience of a territory and the strength of the natural environment. It was clear that all participants experienced a sense of empowerment when looking at this photograph, which represented the resistance of their territory amidst urban landscapes, boldly showcasing the persistence of nature and the power of community action.

"Even though we are imprisoned by the cities that surround us, nature is stronger, so we were taught to take care of it." (Id 16).

"The villages are squeezed by the cities, there we preserve, but all around it is being destroyed, because for them the land is just a piece of land that can bring money. (Id 1)"

The participants’ statements reveal the conflicts that arose from encroaching urban development. One participant viewed the preservation of their land as a symbol of resilience, while another expressed frustration over its misuse, which threatened their ability to fully engage with their culture and the land. This led to a diminished sense of cultural centeredness and exacerbating the inequalities faced by their community. The photograph captured the narrative that, despite the village being increasingly surrounded by buildings, it remained perched upon the city. A recurring theme identified in the analysis was the impact of land demarcations—or the lack thereof. Participants noted that effective demarcations often fail due to a lack of political will, and when they occurred, they involved numerous struggles and bureaucratic negotiations among diverse interests. This process can escalate into violent conflicts between Indigenous and non-Indigenous communities, with Indigenous peoples frequently at a disadvantage due to limited resources.

Another critical aspect of seeking respect for demarcated lands was the understanding of the sensitivity surrounding land leasing and the distinction between land and territory for Indigenous peoples. Participants emphasised that Indigenous land exists independently of demarcation; it is inherent. Through their photographs, they articulate the necessity of land demarcation, which creates protected spaces essential for preserving their traditions and culture.

(2) Humanity's destructive action and the voice of nature

The Indigenous participants voiced their concerns about the long-standing aggression towards nature, and emphasised the accelerated pace of this destruction in recent times.

**Fig. 5:**
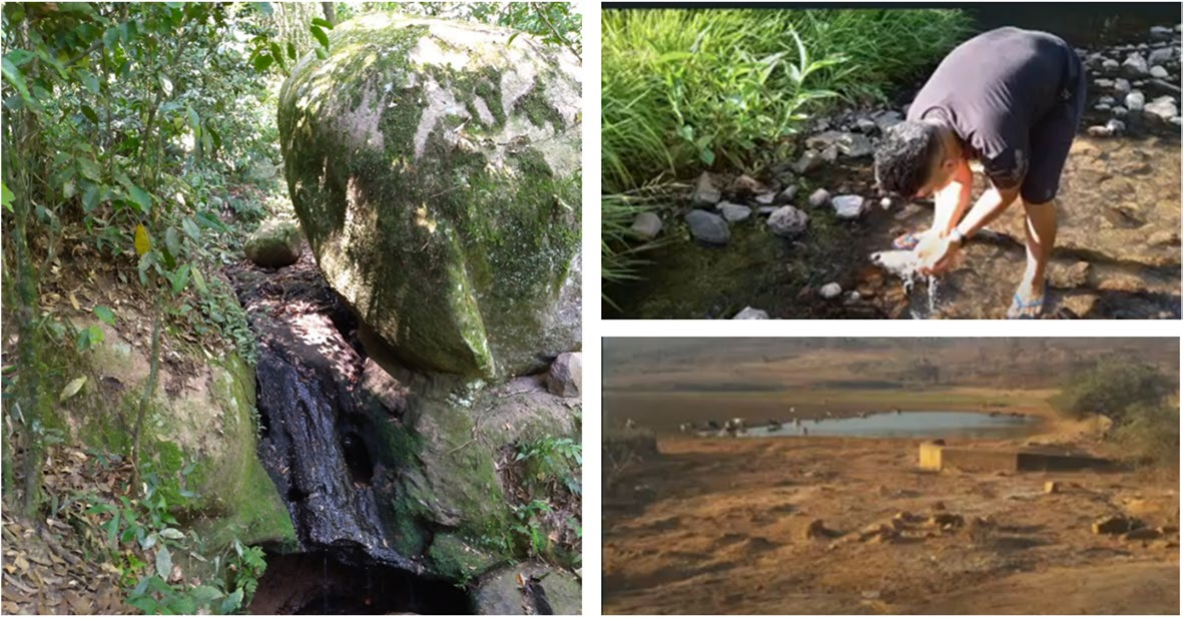
Rivers are polluted and dying

Figure 5 highlights the importance of water, which participants considered as one of the most precious elements of life on Earth. Water is essential for meeting various human needs, including food production and sustaining ecosystems. Several photographs depicted rivers and springs that are drying up or have already dried up, as well as rivers contaminated with chemicals from agriculture and industry.

"The rivers could be full of fish for everyone to feed, but human beings are more concerned with capital than anything else, the river I have known since I was a child has become shallower and shallower." (Id 8).

"In addition to the problem of contamination of the rivers that cross towns and farms along the way, they are drying up. The river I used to swim in now hits the foot." (Id 20).

Participants provided insight into the precariousness of their water supply, as their villages face droughts that severely compromise the food sovereignty of Indigenous peoples, most of whom rely on subsistence farming. They described the provision of drinking water as a longstanding issue that has worsened due to recent climate changes. Some villages have turned to alternative methods of obtaining water, such as tanker trucks, but felt that these were inadequate solutions.

One participant noted that this often forced them to seek water from sources that may not be safe for consumption. Consequently, in addition to food scarcity resulting from the loss of animals due to malnutrition, Indigenous communities express a genuine fear of consuming water, even when it is their only option.

**Fig. 6:**
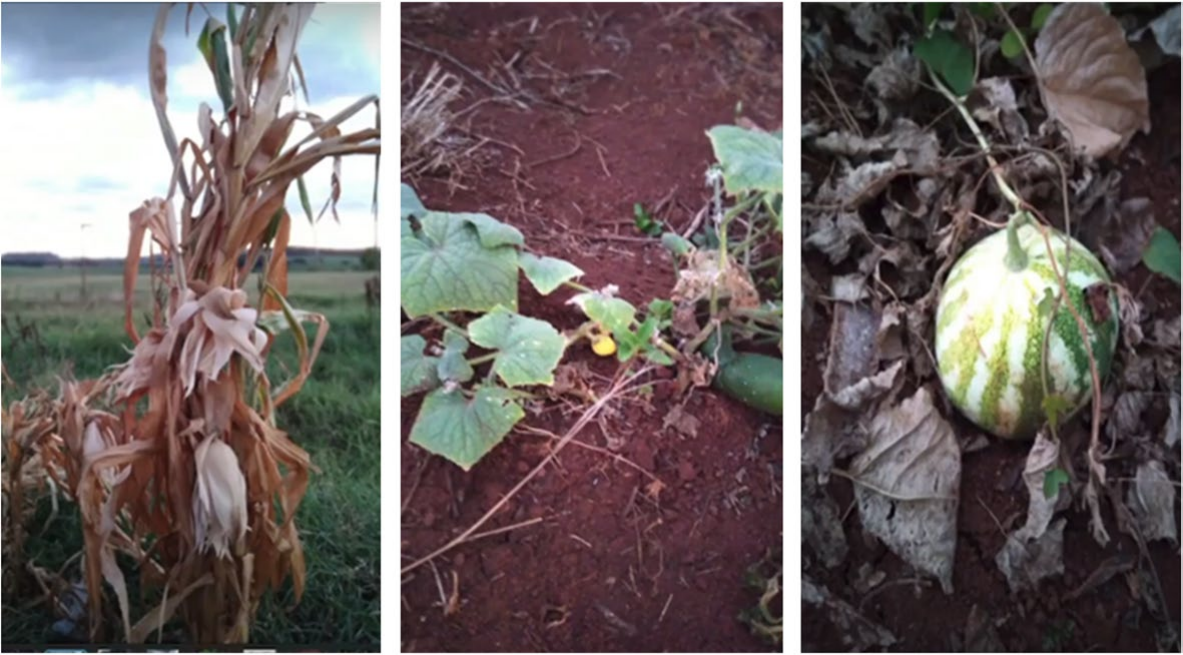
Climate change and the impact on our food

Figure 6 illustrates the impact of climate change on food security.

"Our plantation did not thrive either, the corn dried up, the dam dried up, due to the lack of rain, it was very sad to lose everything." (Id12).

"We even lost the calves, because we didn't have water to give them, the watermelon, the pumpkin, the beans, nothing grew with this drought." (Id 8).

Participants shared a common experience of facing water scarcity during the drought season. Both expressed their despair over the detrimental effects of water shortages on crops and wildlife. Additionally, participants identified waste as another significant consequence of climate change in their daily lives.

Many commented on the excessive production and improper disposal of garbage, which they attributed to rampant consumerism. According to some students, the increasing consumption of products leads to a surge in waste generation. They cautioned that improper waste disposal can cause a range of environmental problems, including contamination of water, soil, and air, as well as economic, social, and health issues.

"They don't help us take care of it, for example, one day of the week is chosen here to do all the cleaning in the village. They (non-Indigenous people) barely take care of their place, let alone a place that belongs to everyone, so many times we get angry, we are trying to take care of the world, but most people don't care." (Id 15).

"We take care of and preserve it, but around people don't do that, it's a disregard for the environment, they leave their garbage on any corner. Is this responsibility just for us?" (Id 1)."

**Fig. 7:**
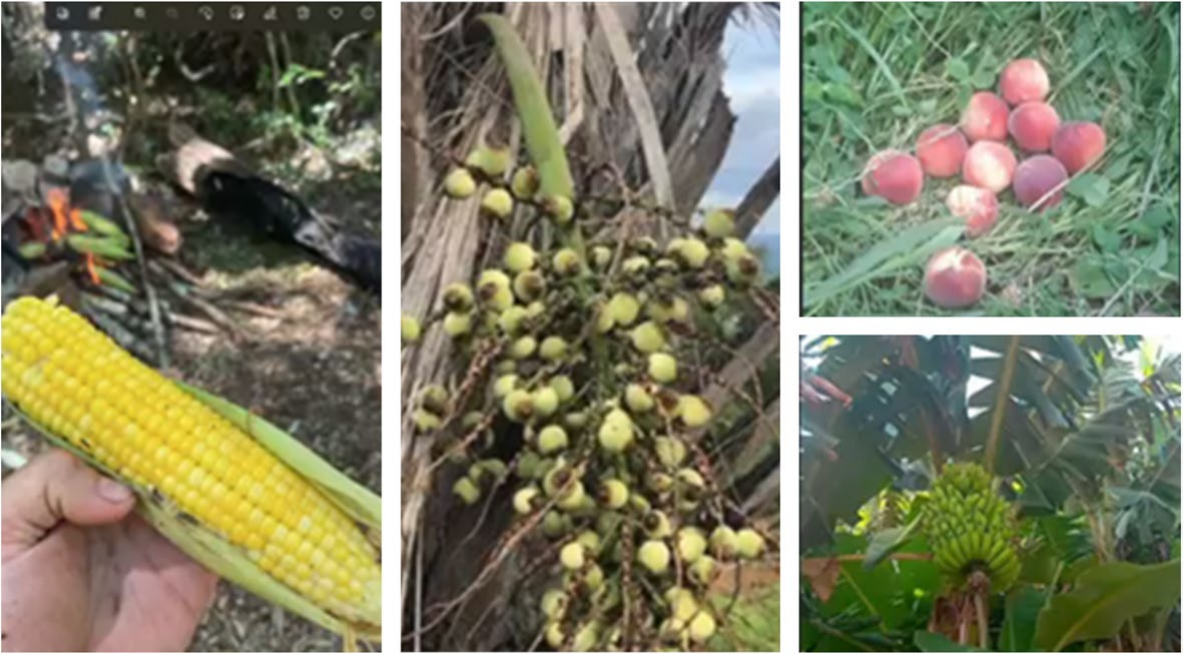
Nature is harmonious and offers everything necessary for survival

Participants expressed deep concern about the harm inflicted on nature and its resulting impact on the environment. They emphasised their cultural commitment to preservation, positioning themselves as crucial stakeholders in mitigating these issues, as they have evolved alongside nature in a profound reciprocal relationship. As custodians of ancestral knowledge, they viewed themselves as guardians of the forest, believing that nature communicated through the winds, rivers, and skies both day and night. They emphasised that nature is harmonious and provides everything necessary for survival. Figure 7 illustrates the harmony of nature and its essential contributions to life.

"Nature speaks to us in many ways, we know when it will rain, where we should fish, where and when we should plant. She gives us everything we need." (Id 9).

"We don't need much, we need our river, our land to have our food. If we know how to respect nature, it will meet our survival needs." (Id 3).

However, they cautioned that the world is changing, and the places they cherished in their childhood no longer exist. Many traditional activities practiced in the village, such as fishing and foraging for fruits in the forest, are compromised. As human actions threaten the survival of various species and jeopardize their health, it becomes increasingly challenging to attune to the rhythms of nature.

"Nothing is like it used to be, the wind doesn't blow like it used to, spiritists aren't guiding us anymore, the voices we used to hear aren't with us anymore, the Great Spirit is asking for help because they just want to take money out of the earth, they don't want to take care of her." (Id. 16).

"Our survival depends on nature. If nature is being destroyed, we are being destroyed too. Now nature is sending us a desperate message for help, and we are running out of time." (Id. 4).

While participants voiced their concerns about the destruction of nature, it is important to emphasise the constructive sentiments expressed in their statements. They highlighted teachings from Indigenous culture that emphasised the possibility of enjoying nature without harming the ecosystem, underscoring that all forms of survival are directly dependent on environmental conservation.

(3) Ancestry protection, knowledge and resiliency of Indigenous culture

This category highlighted the significance that Indigenous Peoples place on their ancestors and their commitment to resisting attacks on their culture. Participants described ancestry as a vital source of life, wisdom, identity, and belonging. Recognising that their values and meanings are deeply rooted in ancestral knowledge—often passed down through generations—many participants characterised ancestry as the thread that interweaves the past, present, and future, creating a web of spiritual relationships. In Indigenous belief, all beings possess a spirit that deserves respect and connection to their ancestry, as ancestors are viewed as protectors, guides, and teachers of reverence for nature. For participants, reflecting on those who came before them is a way of understanding their history and acknowledging a path that has been traced in various ways, as well as remembering their origins, values, and beliefs.

**Fig. 8:**
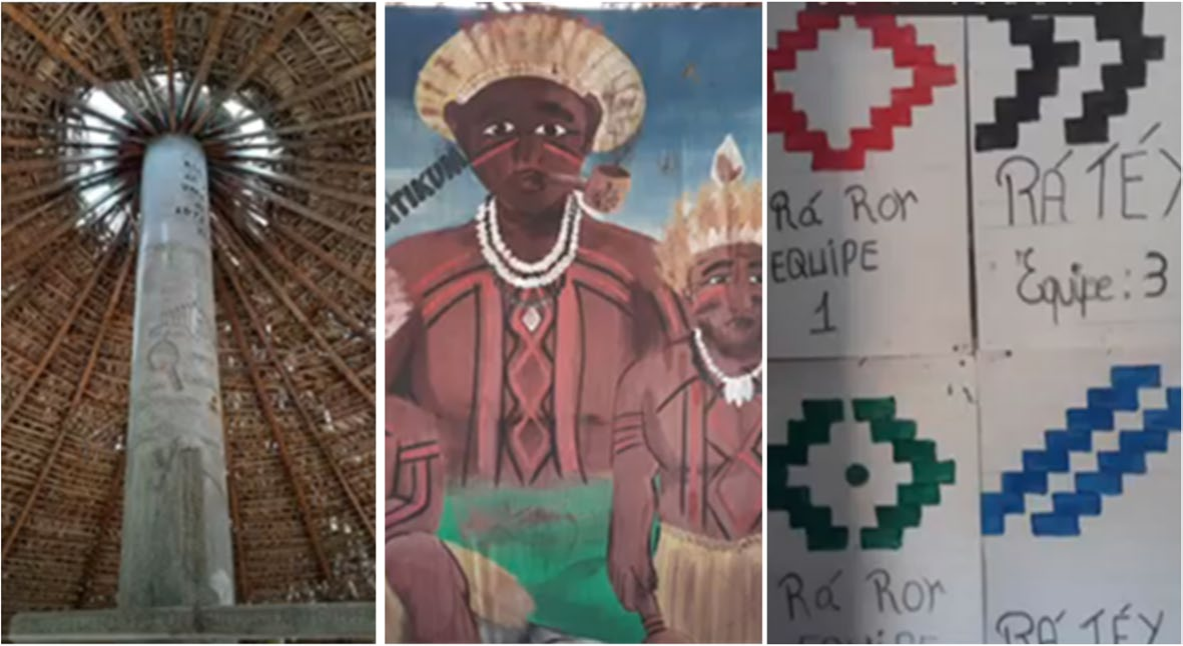
The power of our ancestors does not let them take everything

Figure 8 illustrates the power of their ancestors.

"We are aware that nature is collective property and that ancestral power resists in the midst of it because our ancestors were buried there." (Id 2)

"Despite men advancing and destroying, the power of our ancestors does not let them take everything. There will always be a piece of us, whether in parks, rivers, forests, nature reserves, etc.…" (Id5)

**Fig. 9:**
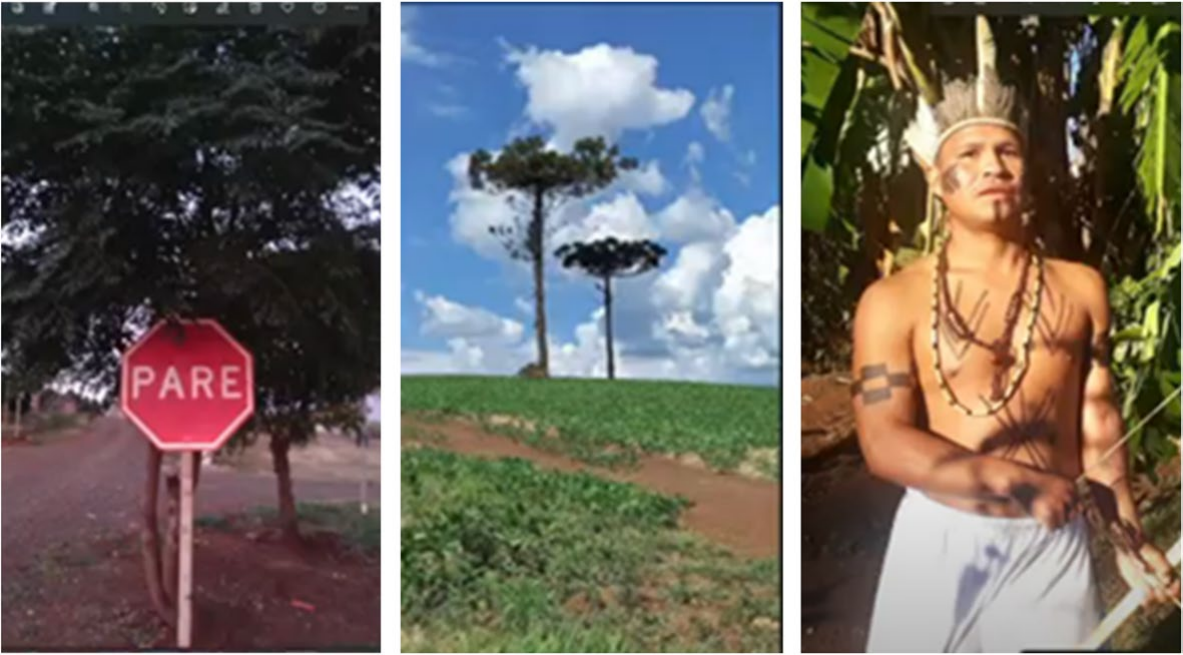
Nature and our ancestors are on our side

In this context of honouring and respecting their ancestors, participants shared that recognizing the immense struggles endured by these individuals to secure greater autonomy today fuels their determination to preserve their culture and ancestral territories. They pointed out the challenges they encountered in a society that often overlooks Indigenous communities, which can lead to displacement from their lands and experiences of humiliation and subjugation. Figure 9 illustrates the collaborative relationship between nature and their ancestors, highlighting how both are integral to their identity and resilience.

"We must continue the fight, we must resist the interests of money that lead to the destruction of our planet. Nature and our ancestors are on our side." (Id 11).

"We will not give up the fight, our ancestors fought and defended the right we have today, so it is our duty to continue fighting as they did." (Id 17).

This stop sign, positioned at the entrance to a participant's village symbolises a call to halt actions detrimental to Indigenous culture—a reminder to *"pause, step back, and acknowledge the significance of this territory".* Similarly, the image of the two Arcaria trees, native to the region, evokes a powerful response. Many of these trees have been felled to make way for expansive soybean fields, yet these two remain standing as symbols of resilience and Indigenous resistance. Participants were eager to showcase a photograph of an individual dressed in traditional attire, representing the ongoing struggle to defend Indigenous culture against external threats.

## Discussion

The findings of this study highlight the critical role that Indigenous university students play in documenting and advocating for their communities through the photovoice methodology. Participants illustrated their experiences and connections to their lands, revealing the intricate relationships between identity, culture, and the environment. The photographs captured not only the beauty of their surroundings but also the challenges posed by climate change, environmental degradation, and encroachment from agribusiness. This aligns with existing literature that emphasizes the importance of Indigenous perspectives in understanding and addressing environmental issues [16, 17].

The narratives shared by participants underscore the urgent need to protect Indigenous lands, which are vital for their cultural survival and resilience. The students articulated a deep understanding of the interconnectedness of all beings, viewing themselves as stewards of the land who possess ancestral knowledge crucial for environmental conservation. Their insights resonate with the growing recognition of Indigenous wisdom in contemporary environmental discourse [18].

Moreover, the study highlights the tensions surrounding land leasing practices, which many Indigenous communities face due to economic pressures. Participants expressed frustration over the challenges in maintaining their cultural practices and accessing natural resources amidst these pressures. The recognition that land demarcation often fails due to political inertia further complicates their efforts to safeguard their territories. As noted by previous research, effective land management requires not only recognition of Indigenous rights but also active involvement in decision-making processes [19].

Despite the grim realities depicted in the participants' narratives, there is a resilient spirit evident in their commitment to cultural preservation and environmental stewardship. The acknowledgment of their ancestors as a source of strength emphasizes the significance of intergenerational knowledge in the fight against climate change. This aligns with findings by other scholars, who argue that Indigenous perspectives are vital for fostering sustainable practices [20].

Indigenous values of reciprocity and stewardship underscore the critical importance of land for global Indigenous communities. Their cultural practices do not center around the consumption of market goods, as they reject the exploitation and depletion of natural resources [21]. Research indicates that the unsustainable habits prevalent in Western society can lead to rapid resource depletion and significant environmental contamination [22].

Water scarcity is a pressing concern for Indigenous peoples, who actively strive to conserve clean water sources and rehabilitate damaged springs. They understand that without such protection, water availability will decrease, watercourses may dry up, and the overall quality of this vital resource will be compromised, negatively impacting all living beings reliant on it for survival. Indigenous communities have long upheld a harmonious relationship with the environment, recognizing it as integral to their way of life. Thus, the preservation of their territories is fundamental for sustaining life within their communities.

The collective spirit embedded in Indigenous culture offers valuable lessons for other societies, particularly regarding respect for ancestors and the ongoing struggle to preserve cultural identity. Moreover, much of Brazil's cultural heritage has been inherited from Indigenous peoples, whose contributions to vocabulary, cuisine, music, and dance are immeasurable. However, there remains a critical need for greater respect and acknowledgment of this ancient culture and its wisdom, which can provide valuable insights for future generations.

### Study strengths and limitations

Participants were Indigenous university students, and this would have influenced the narratives. As a minority group at university, Indigenous students navigate dual identities, balancing their traditional community roles with the expectations of academia. This involves cultural negotiation, where students serve as cultural mediators, advocating for Indigenous representation in academic spaces and simultaneously taking knowledge from the university to their communities. Examples include organising cultural exchange events, building academic networks to challenge stereotypes, and using their unique position to foster dialogue and mutual understanding. They also actively challenge Eurocentric academic narratives by asserting Indigenous perspectives and a desire for knowledge to be rooted in Indigenous epistemologies. Many used social media platforms to amplify their voices, sharing stories of resilience and mobilizing support for Indigenous rights. Photovoice engaged students in Indigenous knowledge creation about issues that they advocated for. The arts-based narratives are powerful but some limitations should be noted.

First, the focus on specific regions (Mato Grosso do Sul and Rio Grande do Sul) may limit the generalizability of the findings to other Indigenous communities in Brazil or beyond, as cultural practices and environmental issues can vary significantly across regions. Secondly, the reliance on photovoice as a primary data collection method may pose challenges related to representation and interpretation, as participants may have had different levels of comfort and skill in using photography to express themselves. The online format of workshops during the COVID-19 pandemic could have restricted engagement, affecting the depth of interactions among participants. Lastly, the study's cross-sectional design captured perspectives at a single point in time, and longitudinal research would be valuable to understand how Indigenous communities navigate changing environmental conditions and socio-political landscapes over time. Future studies could benefit from incorporating a broader range of Indigenous voices and exploring their experiences of multiple and intersectional risks.

### Implications and recommendations

The findings of this study underscore the importance of integrating Indigenous perspectives into broader academic and policy discourses. Some key issues that were strongly emphasised in the narratives included the need for (i) integrating Indigenous knowledge in environmental policy frameworks, and ensuring that their voices are represented in decision-making processes related to land management and environmental conservation; (ii) greater advocacy efforts to protect Indigenous territories from exploitation. This included effective land demarcation processes and policies that prioritise the environmental and cultural integrity of these lands and (iii) recognition that the principles of reciprocity, stewardship, and sustainability exemplified by Indigenous communities can be valuable for tackling global environmental challenges. More broadly the narratives, sensitive and articulate, from the Indigenous university student signalled the important role of universities in capacity building Indigenous students and ensuring curricula that are inclusive of Indigenous epistemologies, allowing Indigenous students to see their traditions and perspectives reflected in academic spaces. Universities can act as critical links between Indigenous communities and policymakers, facilitating Indigenous voices for environmental protection. The urgency for action on issues raised by the Indigenous students cannot be overstated, as they directly impact not only Indigenous self-determination but also the broader global struggle against environmental degradation. Recognising and supporting the roles of Indigenous peoples as environmental stewards is an essential step toward a more sustainable and equitable future.

## Conclusion

This study highlighted the intrinsic relationship between Indigenous peoples and nature, emphasising the urgent need to protect their territories and respect their cultural identities for their survival and the preservation of their traditions. Achieving this requires intersectoral actions that address the macro-level challenges while engaging directly with Indigenous communities to understand their stories and implement culturally sensitive, sustainable solutions. As climate change and environmental adversities continue to take center stage, promoting Indigenous culture can help raise awareness about the importance of sustainable living and the conservation practices rooted in millennia of knowledge. This research contributes to the growing body of literature on Indigenous methodologies and "two-eyed" seeing, focusing on themes such as knowledge, creativity, respect for nature, and ancestral ties, all vital for a healthier environment.

Despite its successes, the study faced limitations, including cultural differences, COVID-19 restrictions, and the inability to conduct face-to-face meetings. Nonetheless, the co-production process fostered critical reflection among participants and researchers, leveraging arts-based methods for advocacy. The resulting archive of images, with proper authorisations for use, serves as a valuable resource for future culturally sensitive communications with local communities and for ongoing research dissemination.

## Data Availability

The datasets generated and/or analysed during the current study are not publicly available to protect study participant privacy, but are available from the corresponding author on reasonable request.
